# Systematic Fine-Mapping of Association with BMI and Type 2 Diabetes at the *FTO* Locus by Integrating Results from Multiple Ethnic Groups

**DOI:** 10.1371/journal.pone.0101329

**Published:** 2014-06-30

**Authors:** Koichi Akiyama, Fumihiko Takeuchi, Masato Isono, Sureka Chakrawarthy, Quang Ngoc Nguyen, Wanqing Wen, Ken Yamamoto, Tomohiro Katsuya, Anuradhani Kasturiratne, Son Thai Pham, Wei Zheng, Yumi Matsushita, Miyako Kishimoto, Loi Doan Do, Xiao-Ou Shu, Ananda R. Wickremasinghe, Hiroshi Kajio, Norihiro Kato

**Affiliations:** 1 Department of Gene Diagnostics and Therapeutics, Research Institute, National Center for Global Health and Medicine, Tokyo, Japan; 2 Department of Public Health, Faculty of Medicine, University of Kelaniya, Ragama, Sri Lanka; 3 Bach Mai Hospital, Hanoi, Vietnam; 4 Division of Epidemiology, Department of Medicine, Vanderbilt Epidemiology Center, Vanderbilt-Ingram Cancer Center, Vanderbilt University School of Medicine, Nashville, Tennessee, United States of America; 5 Division of Genomics, Medical Institute of Bioregulation, Kyushu University, Fukuoka, Japan; 6 Department of Clinical Gene Therapy, Osaka University Graduate School of Medicine, Suita, Japan; 7 Department of Geriatric Medicine and Nephrology, Osaka University Graduate School of Medicine, Suita, Japan; 8 Center for Clinical Sciences, National Center for Global Health and Medicine, Tokyo, Japan; 9 Center Hospital, National Center for Global Health and Medicine, Tokyo, Japan; National Cerebral and Cardiovascular Center, Japan

## Abstract

**Background/Objective:**

The 16q12.2 locus in the first intron of *FTO* has been robustly associated with body mass index (BMI) and type 2 diabetes in genome-wide association studies (GWAS). To improve the resolution of fine-scale mapping at *FTO*, we performed a systematic approach consisting of two parts.

**Methods:**

The first part is to partition the associated variants into linkage disequilibrium (LD) clusters, followed by conditional and haplotype analyses. The second part is to filter the list of potential causal variants through trans-ethnic comparison.

**Results:**

We first examined the LD relationship between *FTO* SNPs showing significant association with type 2 diabetes in Japanese GWAS and between those previously reported in European GWAS. We could partition all the assayed or imputed SNPs showing significant association in the target *FTO* region into 7 LD clusters. Assaying 9 selected SNPs in 4 Asian-descent populations—Japanese, Vietnamese, Sri Lankan and Chinese (*n*≤26,109 for BMI association and *n*≤24,079 for type 2 diabetes association), we identified a responsible haplotype tagged by a cluster of SNPs and successfully narrowed the list of potential causal variants to 25 SNPs, which are the smallest in number among the studies conducted to date for *FTO*.

**Conclusions:**

Our data support that the power to resolve the causal variants from those in strong LD increases consistently when three distant populations—Europeans, Asians and Africans—are included in the follow-up study. It has to be noted that this fine-mapping approach has the advantage of applicability to the existing GWAS data set in combination with direct genotyping of selected variants.

## Introduction

Genome-wide association studies (GWAS) have succeeded in identification of a number of genomic loci that are associated with complex diseases and traits. However, the loci identified through GWAS often extend over hundreds of kilo-bases (kb) and contain many genes and a large number of genetic variants with indistinguishable signals of association, occurring as a result of linkage disequilibrium (LD), i.e., non-random coinheritance of genetic variants, across the region. A set of SNPs in strong LD are inherited together by forming haplotypes. Given that the SNPs on GWAS chips are “tags” for haplotypes on which the unobserved functional variants may reside, the major challenge emerging from GWAS is to refine the location of causal variants within established loci, which is called fine mapping, in order to prioritize genes for follow-up through functional studies [1].

To date, a substantial proportion of GWAS have been performed in populations of European descent, which tend to have longer haplotype blocks containing a larger number of highly correlated SNPs than non-European populations [2], hampering the identification of causal variants, subsequent to GWAS. Under these circumstances, it has been proposed by several groups that trans-ethnic meta-analysis improves the resolution of fine mapping at some loci if the same SNPs could show association concordantly across different populations [3]–[5]. This supports the tenet that the same causal variant(s) should exist at such an associated locus in the different populations. Extensive simulations of fine-mapping studies have shown that the average power to resolve the causal SNPs from those in strong LD increases consistently when multiple distant populations are genotyped in the follow-up study, as compared to the cases of genotyping any single population [3].

In terms of trans-ethnic meta-analysis and fine-mapping, a few groups have thus far examined the association between genetic variants at the fat mass and obesity associated (*FTO*) gene locus on 16q12.2 and body mass index (BMI) by simply comparing the list of significantly associated SNPs in African Americans with those previously reported in Europeans [6], [7]; they have claimed that the use of populations with low average LD, such as African-descent populations, results in the greater resolution of fine mapping at *FTO*. In the present study, we have tested whether integrating results from multiple ethnic groups (including a total of 26,109 and 24,079 subjects of Asian ancestry for BMI and type 2 diabetes associations, respectively) could further improve the resolution of fine-mapping at *FTO* by performing a systematic approach, which involves cross-population filtering of potential causal variants, following stepwise conditional analysis and haplotype analysis in order to reduce the number of candidates.

## Materials and Methods

### Ethics Statement

All human participants provided written informed consent, and the ethics committees of the National Center for Global Health and Medicine (NCGM), Kyushu University, Osaka University, University of Kelaniya, Bach Mai Hospital, and Vanderbilt University approved the protocols.

### Study populations

We performed an association study of genetic variants at *FTO* with BMI and type 2 diabetes in four Asian-descent populations—Japanese, Vietnamese, Sri Lankan and Chinese populations. Details about individual populations are provided in Table S1.

#### Japanese populations

For BMI association study, subjects from two general Japanese populations were included in the present study. Specifically, 5,779 Japanese participants (the Amagasaki panel) were consecutively enrolled in the population-based setting as described previously [8] and 12,569 participants (the Fukuoka panel) were randomly selected from residents aged 50 to 74 years in the general population [9], from which 4,880 non-diabetic subjects were chosen to assess diabetes-independent genetic impacts on BMI. For type 2 diabetes association study, 5,252 cases and 6,156 controls, who were enrolled from the Cardiovascular Genome Epidemiology (CAGE) Network [10], were examined. From the CAGE Network, type 2 diabetes cases were enrolled according to the WHO criteria, while unaffected controls were enrolled according to the following criteria: no past history of urinary glucose or glucose intolerance; HbA_1c_ <6.0% or a normal result from 75 g glucose tolerance test; and age at examination ≥55 years. Among the Japanese subjects, there was some overlap between the BMI and type 2 diabetes studies.

#### Vietnamese populations

For BMI association study, subjects from general Vietnamese populations were examined. The participants were recruited to longitudinally identify the prevalence and incidence of diabetes and metabolic syndrome-related diseases at two separate sites of Vietnam—the capital (Hanoi) and lowland province (Thai Binh)—in a population-based setting. A total of 3,782 subjects (1,616 in Hanoi and 2,216 in Thai Binh) were enrolled at a baseline survey between January 2008 and September 2009. For type 2 diabetes association study, 281 cases and 915 controls were selected within the cohorts; diabetes was defined as fasting plasma glucose level ≥7.0 mmol/l and/or under treatment for type 2 diabetes, and the controls were chosen as non-diabetic participants who met the following conditions: plasma levels of fasting glucose <6.1 mmol/l and 2-hr postprandial glucose <7.77 mmol/l; no previous and current treatment for diabetes; and age ≥55 years.

#### Sri Lankan populations

For BMI association study, subjects from general Sri Lankan populations were examined. The participants were recruited at two separate sites of Sri Lanka—the urban area (Ragama) near Colombo and tea plantation estates near Nuwara Eliya (180 km from Colombo)—in a population-based setting [11], [12]. A total of 3,041 subjects aged 35–64 yrs (2,691 in Ragama and 350 in estates) were included in this study. For type 2 diabetes association study, 714 cases and 1,780 controls were selected within the cohorts; diabetes was defined as fasting plasma glucose level ≥7.0 mmol/l and/or under treatment for type 2 diabetes, and the controls were chosen as non-diabetic participants who met the following conditions: plasma levels of fasting glucose <6.1 mmol/l and HbA_1c_ <6.0%; and no previous and current treatment for diabetes.

#### Chinese populations

For BMI association study, 8,981 participants with complete BMI and genotyping information from the Shanghai Genome-Wide Association Studies (SGWAS) were examined. SGWAS included participants of the Shanghai Breast Cancer Study (SBCS), Shanghai Endometrial Cancer Study (SECS), Shanghai Breast Cancer Survival Study (SBCSS), Shanghai Women' Health Study (SWHS), and Shanghai Men's Health Study (SMHS) [13]. The SBCS and SECS are population-based case-control studies, and the SBCSS, SWHS and SMHS are ongoing population-based, prospective cohort studies. All participants of these studies were recruited in Shanghai using similar study protocols. For type 2 diabetes association study, we identified 1,123 cases among the total of 8,981 participants, in which the remaining 7,858 participants were served as controls [14].

In this article, we use a term, East Asian, when the samples include Japanese, Vietnamese and Chinese, and use a term, Asian, when the samples also include Sri Lankans. Japanese, Vietnamese and Sri Lankan populations were subjected to SNP–trait association analysis, conditional analysis and haplotype analysis for BMI and type 2 diabetes, whereas Chinese populations were subjected to haplotype analysis alone. In Japanese, among 5,252 cases used for type 2 diabetes association study, 66 individuals overlapped with 5,779 participants in the Amagasaki panel, which was used for BMI association study; i.e., the remaining 5,186 cases were not included in the BMI association study. In the other Asian-descent populations, type 2 diabetes cases were selected within the cohorts and hence included in the BMI association study.

### SNP genotyping and quality control

First, to investigate LD relations between SNPs showing strong association signals at *FTO* on 16q12.2, we used part of the GWAS data for type 2 diabetes (*n* = 2,046) in the Japanese case-control study panel. In the genome-wide association scans, genotyping was performed with bead arrays (Infinium HumanHap550 and HumanHap610-Quad; Illumina, San Diago, CA, USA) and quality control was done as previously described [10]. Imputation of genotypes was carried out to the HapMap Phase 2 data set (hapmap.ncbi.nlm.nih.gov/) and to the 1000 Genomes Project data set (http://www.1000genomes.org/) with metric for imputation accuracy set at *Rsq*>0.5.

Then, we genotyped Asian samples (except for the SGWAS panel) using the TaqMan assay (Applied Biosystems by Life Technologies, Carlsbad, CA) for 9 SNPs at *FTO*, which were chosen as target variants by trans-ethnic fine-mapping mentioned below. The genotype distribution of all tested SNPs was in Hardy–Weinberg equilibrium (*p*>0.01). We obtained successful genotyping call rates of >99.4% for all SNPs and >99.7% for all included samples (across 9 SNPs).

Chinese samples in the SGWAS were genotyped with the Affymetrix Genome-Wide Human SNP array 6.0 and quality control was done as previously described [13].

### Statistical analysis

#### SNP–trait association analysis

To calculate BMI, each study collected weight and height measurements. To improve the normality of the BMI distribution and alleviate the impact of outliers, the rank-based inverse normal transformation was applied to BMI values separately for each gender in each study as previously reported [13]. The association between SNPs and the inverse normal transformed BMI values was analyzed in a linear regression model with adjustment for age and age-squared. Also, association between SNPs and type 2 diabetes was tested by logistic regression in an additive model with adjustment for sex.

For testing each SNP–trait association, a *p-*value of <0.05 was considered statistically significant, where, for an association to be considered significant, it had to involve the same risk (i.e., BMI-increasing and disease risk) allele as that reported in European-descent populations [15]. We combined association results for populations in each ethnic group and across different ethnic groups by using the inverse-variance weighting method. We used PLINK (http://pngu.mgh.harvard.edu/~purcell/plink/), the R software (version 2.8.1; www.r-project.org), the rmeta package (http://cran.r-project.org) and the METASOFT (http://genetics.cs.ucla.edu/meta/) for association test and meta-analysis. We examined the heterogeneity of the per-allele effect size between the ethnic groups with Cochran's Q-test.

#### Index SNPs for fine mapping

**Figure 1:**
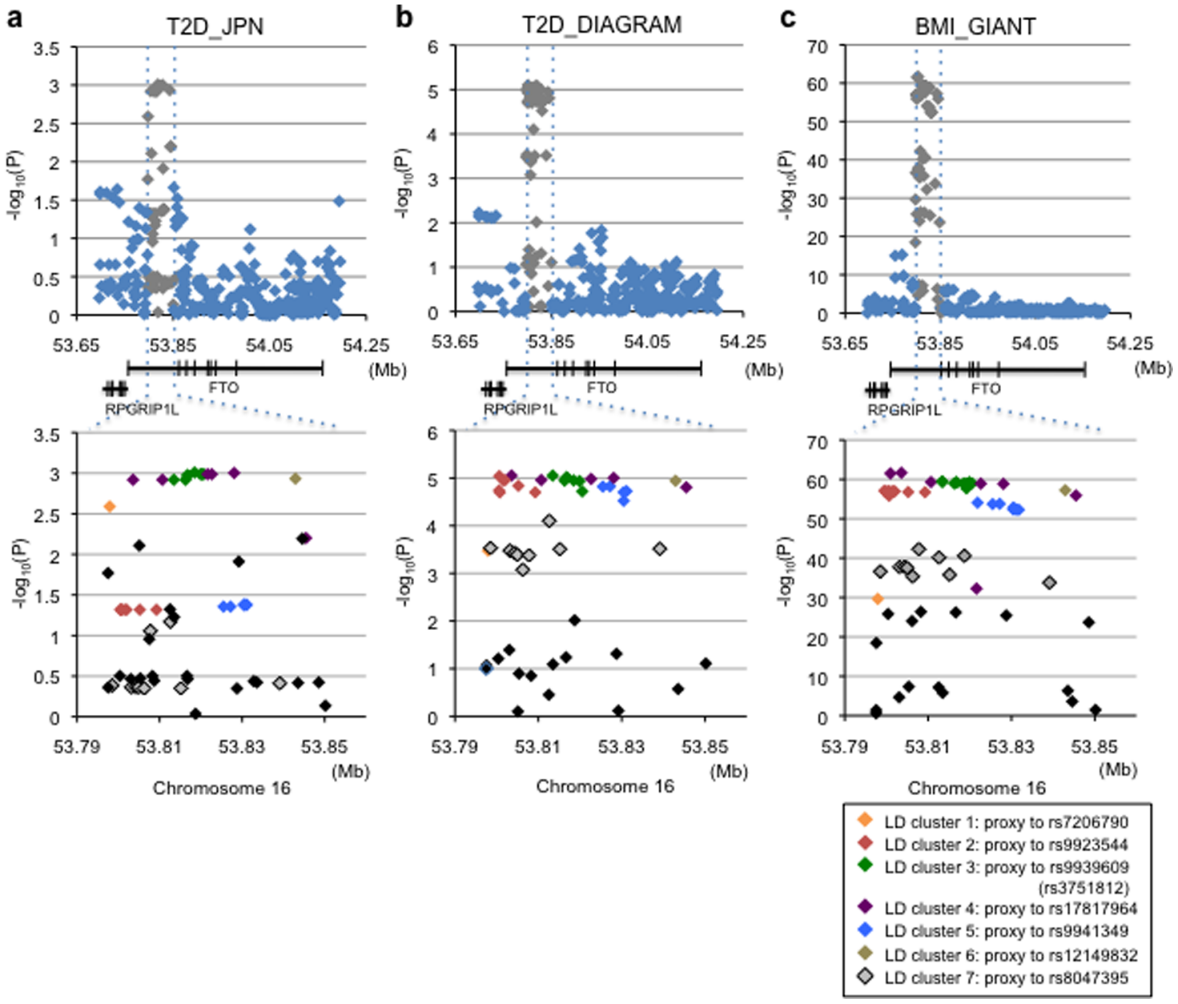
Plots of SNP–trait association and SNP partitioning for the 16q12.2/*FTO* region in Japanese (type 2 diabetes, a) and Europeans (type 2 diabetes, b; BMI, c). Association results for Europeans are drawn from the published studies [19], [20]. a, b and c each contain three panels. In the top panels, all assayed/imputed SNPs in the GWA scan (that passed the quality control) are plotted with their −log10 (*p-*values) for type 2 diabetes (a and b) and BMI (c) against chromosome position (in Mb): genotypes are imputed to the HapMap Phase 2 data set. In the second panels, the genomic locations of RefSeq genes with intron and exon structure (NCBI Build 37) are displayed. The third panels show the plots for the intron-1 *FTO* region, where the associated SNPs are partitioned into seven clusters and colored accordingly (see Methods).

It is possible that there are >1 causal variants in the region of interest, in the cases such as allelic heterogeneity. Therefore, we extracted a list of ‘top hit’ (or index) SNPs that could represent an independent association among the association signals, which were shown to be clustered at *FTO* (Text S1). Briefly, after close inspection of the LD relationship between SNPs shared by multiple ethnicities, we first selected a list of index SNPs that satisfied a certain level of statistical significance, in particular, referring to European data, such that they belonged to independent LD clusters with each other at a given LD coefficient level. We then partitioned all the assayed/imputed SNPs showing significant association in the intron-1 of *FTO* (Figure 1) so that those in stronger LD with one index SNP (e.g. SNP-A) than with another index SNP (e.g. SNP-B) were grouped into a bin of SNP-A's correlates. Here, the number of bins is equal to the number of selected index SNPs, which is largely dependent on the level of LD coefficient, *r^2^*. With a lower *r^2^* threshold (e.g., 0.8 or 0.5), the number of LD clusters becomes smaller, while each LD cluster contains a larger number of SNPs; this is not suitable for fine mapping. On the other hand, with an excessively high *r^2^* threshold (e.g., 0.95), the phenotype association of index SNPs, mutually in such high LD, becomes indistinguishable by conditional analysis using a realistic number of samples. Thus, we adopted an *r^2^* threshold of 0.9 (Figure S1), which was assumed to be appropriate for fine mapping at *FTO* with the sample size tested in the present study (see Text S1).

#### Test of independent associations

We performed a stepwise multiple regression analysis (or conditional analysis) to see whether there were >1 causal variants in the region of interest and to test which was most likely to explain the signal of association among a set of SNPs in modest LD with each other. That is, if SNP-A remains significant (*p*<0.05 after adjustment for multiple testing) with SNP-B included in the regression model, but SNP-B is not significant with SNP-A in the regression model, then SNP-A is more likely to be causal or more closely correlated with the causal variant. On the other hand, if two SNPs simultaneously included in the model each attain significance, they may have independent associations.

#### Haplotype analysis

For fine mapping at *FTO*, we selected index SNPs aforementioned or significant SNPs previously reported in European and African American studies [6], [7], [15], inferring the haplotypes using the PLINK [16] and PHASE [17] softwares (http://depts.washington.edu/ventures/UW_Technology/Express_Licenses/PHASEv2.php). We then tested which haplotypes were strongly associated with the trait. In parallel, haplotypes were inferred from the genotype of SNPs in the 1000 Genomes Project (www.1000genomes.org) data for Phase-1 samples—East Asian Ancestry (ASN), European Ancestry (EUR) and African Ancestry (AFR)—using the HaploView software [18].

#### Cross-population filtering of causal variants

To reduce the list of potential causal variants, we closely inspected subsets of SNPs and haplotypes shared by multiple ethnicities. We partitioned all the HapMap SNPs located in the associated region of *FTO* (Figure 1) into subsets (i.e., LD clusters) and then reduced the list of target SNPs by examining a subset (or subsets) of variants that could show a consistent pattern of trait association across different ethnic groups, as previously reported [10].

## Results

### Selection of index SNPs for fine mapping

After close inspection of the LD relationship between *FTO* SNPs showing significant associations with type 2 diabetes in Japanese as well as those with type 2 diabetes and BMI in the European-descent populations (Figure 1), we selected a list of index SNPs that satisfied a certain level of statistical significance (see the details in Text S1). For both phenotype traits concordantly, all the assayed or imputed SNPs showing significant associations in the target region could be partitioned into seven LD clusters, which the selected index SNPs individually represented. In Europeans, where the LD was found to be most conserved among three major ethnic groups (Figure S2), seven LD clusters could be differentiated, whereas two pairs of LD clusters—LD clusters 2 and 5 and LD clusters 3 and 4—were not well differentiated based on the LD coefficient (*r^2^* needed to be set at >0.96) in East Asians (Figure S1). With regard to associations of index SNPs, rs8047395 showed no evidence of type 2 diabetes association in Japanese (*p* = 0.412, Figure 1); hence, LD cluster 7 was excluded from trans-ethnic fine-mapping.

We then tested in Asians the SNP–trait associations at *FTO* for 9 assayed SNPs including 5 index SNPs (apart from an LD-cluster-2 index SNP, rs9923544, which is in complete LD with an LD-cluster-5 index SNP, rs9941349, in Japanese) and those with promising association signals previously reported in African Americans [7]. For both phenotype traits, highly significant *p*-values (*p* = 1.2×10^−7^ for BMI and *p* = 4.8×10^−10^ for type 2 diabetes) were attained for rs1421085, which belonged to LD cluster 3, while the other assayed SNPs except for rs9941349 showed almost equivalent levels of significant association in Asians (Table 1 and Table S2). The assayed SNPs were subjected to conditional analysis. For BMI association, rs9941349 was less closely correlated with the causal variant(s) than any of the others at an adjusted significance level of *p*<0.0056 (≈0.05/9 SNPs; Table S3). In accordance with this, although the statistical significance was borderline (*p* = 0.009) for a single SNP rs17817964, for type 2 diabetes association, rs9941349 was less closely correlated with the causal variant(s) than the other tested SNPs (Table S4). Thus, rs9941349 and its complete proxy rs9923544 (*r^2^* = 1.000 in HapMap JPT+CHB), representing LD clusters 5 and 2, respectively, were also excluded from the list of top hit SNPs in Asians and from further trans-ethnic fine mapping. Similarly, although it did not reach the adjusted significance level, an LD-cluster-1 index SNP, rs7206790 was less closely correlated with the causal variant(s) than any of the others (except for rs9941349) at a nominal significance level (*p*<0.05) for BMI association (Table S3). This is not the case with type 2 diabetes association of rs7206790 (Table S4).

**Table 1:** SNP–BMI association at the *FTO* locus in multiple ethnic groups.

Group		SNPs tested for BMI association at *FTO*								
		rs7206790	rs1421085	rs9939609	rs9941349	rs56137030	rs62033408	rs17817964	rs7188250 (rs72805612)	rs12149832
	Effect/other allele	G/C	C/T	A/T	T/C	A/G	G/A	T/C	C/T	A/G
Japanese (n = 10,659)	Effect al. freq.	0.212	0.183	0.184	0.210	0.183	0.182	0.216	0.189	0.189
	BETA (SE)	0.056 (0.016)	0.072 (0.017)	0.067 (0.017)	0.049 (0.016)	0.068 (0.017)	0.065 (0.018)	0.059 (0.016)	0.064 (0.017)	0.061 (0.017)
	*P*	5.5E-4	2.3E-5	8.5E-5	0.003	6.9E-5	2.1E-4	2.3E-4	2.3E-4	2.5E-4
Vietnamese (n = 3,324)	Effect al. freq.	0.192	0.188	0.189	0.237	0.188	0.187	0.201	0.197	0.192
	BETA (SE)	0.056 (0.031)	0.039 (0.027)	0.051 (0.028)	0.024 (0.026)	0.047 (0.028)	0.051 (0.027)	0.049 (0.028)	0.059 (0.027)	0.067 (0.027)
	*P*	0.072	0.150	0.064	0.364	0.085	0.065	0.073	0.029	0.012
Sri Lankan (n = 3,029)	Effect al. freq.	0.345	0.343	0.328	0.399	0.328	0.326	0.325	0.365	0.368
	BETA (SE)	0.059 (0.027)	0.098 (0.031)	0.1 (0.031)	0.08 (0.028)	0.096 (0.031)	0.098 (0.031)	0.087 (0.03)	0.079 (0.03)	0.071 (0.031)
	*P*	0.033	0.002	0.001	0.005	0.002	0.002	0.004	0.009	0.019
Combined (n = 17,584)	BETA (SE)	0.057 (0.013)	0.069 (0.013)	0.069 (0.013)	0.049 (0.012)	0.068 (0.013)	0.068 (0.013)	0.062 (0.013)	0.065 (0.013)	0.064 (0.013)
	*P*	8.9E-6	1.2E-7	1.3E-7	7.2E-5	2.0E-7	3.7E-7	8.4E-7	6.2E-7	5.7E-7
	*P*_hetero_	0.85	0.65	0.76	0.61	0.80	0.80	0.95	0.89	0.84
European* (n≤123,864)	Effect al. freq.	0.51	0.46	0.46	0.47	N/A	0.44	0.44	0.45	0.45
	BETA (SE)	N/A	0.082 (0.004)	N/A	N/A	N/A	N/A	N/A	N/A	N/A
	*P*	2.3E-30	3.1E-62	9.9E-60	1.8E-54	N/A	N/A	1.4E-59	N/A	5.6E-58
African American (n = 20,488)	Effect al. freq.	0.59	0.12	0.52	0.19	0.12	0.11	0.12	0.12	0.12
	BETA (SE)	N/A	0.053 (0.015)	0.002 (0.009)	0.033 (0.012)	0.064 (0.014)	0.049 (0.015)	0.049 (0.014)	0.064 (0.015)	0.044 (0.015)
	*P*	N/A	3.0E-04	0.82	0.005	8.3E-06	0.001	8.6E-04	1.3E-05	2.5E-03

In each group, two separate panels were characterized and combined by meta-analysis: Amagasaki and Fukuoka panels in Japanese, Hanoi and Thai Binh panels in Vietnamese, and Ragama and estate panels in Sri Lankans (see Methods).

BETA and SEM of each trait are shown as z-score after adjustment for standard deviation.

Besides the SNPs listed in Table 2, two SNPs—rs62033408 and rs72805612 (in complete LD with rs7188250)—were tested for BMI association, because these showed significant association in African-Americans [7].

*P*_hetero_; P-value for Inter-population heterogeneity among three Asian groups.

rs9939609 is in complete LD (*r^2^* = 1.000) with rs62033400 in both HapMap CEU and HapMap JPT+CHB, whereas they are not in strong LD (*r^2^* = 0.065) in HapMap YRI. In African Americans, rs62033400 showed strong association with BMI (*P* = 1.1E-5). In HapMap JPT+CHB, rs9923544 and rs9941349 are in complete LD (*r^2^* = 1.00).

Results for Europeans are drawn from the GIANT consortium (http://www.broadinstitute.org/collaboration/giant/index.php/GIANT_consortium) and those for African Americans are from the published data [7]; the effect allele frequencies in Europeans are from HapMap CEU data.

*In European populations, the results for a proxy, rs1558902, which is in complete LD (*r^2^* = 1.0) with rs1421085 in HapMap CEU, were reported.

When the association results for genotypes imputed to the HapMap Phase 2 data set (Figure 1) were compared with those for genotypes imputed to the 1000 Genomes Project data set (Figure S3), there were no additional, independent association signals, i.e., no additional LD clusters, for type 2 diabetes in Japanese.

Based on the LD coefficients between 9 assayed SNPs, the LD in the target *FTO* region appeared to be less conserved in Asians, as compared to Europeans (Figure S2).

### Haplotype explaining index association

Besides testing single SNP associations, we constructed haplotypes from the SNPs and examined which haplotypes were strongly associated with the trait. On the assumption that haplotypes showing large effects were likely to be correlated with the causal variant(s), we performed haplotype analysis in the combined sample from populations of Asian ancestry, who had the complete SNP data set for haplotype construction (17,128 subjects for BMI and 15,098 subjects for type 2 diabetes; Table 2).

**Table 2:** *FTO* Haplotypes involving index SNPs and their association with BMI and type 2 diabetes.

	Lead SNPs in the associated *FTO* region								Haplotype frequency						BMI association in meta-analysis (n = 17,128)				T2D association in meta-analysis (n = 15,098)			
Haplotype class	rs7206790	rs1421085*	rs3751812	rs9939609	rs9941349	rs56137030*	rs17817964	rs12149832	African ancestry (n = 246)	European ancestry (n = 379)	East Asian ancestry (n = 286)	Japanese (n = 2,046)	Vietnam-ese (n = 1,196)	SriLankan (n = 2,437)	Beta	Standard error	*P*	Hetero-geneity, *P* **	OR	Standard error	*P*	Hetero-geneity, *P* **
H1	G/C	C	T	A	T	A	T	A	0.05	0.41	0.15	0.18	0.18	0.32	0.064	0.014	2.1E-6	0.758	1.221	0.029	8.9E-12	0.695
H1-2	G	C	T	**A**	T	**G**	T	A	None	0.003	None	None	None	Low	−	−	−	−	−	−	−	−
H1-3	G	C	T	**A**	T	A	T	**G**	None	0.01	Low	0.01	0.01	Low	0.048	0.087	0.583	0.211	1.040	0.189	0.837	0.998
H2-1	G	**T**	**G**	**A**	**C**	A	**C**	**G**	0.01	None	None	None	None	None	−	−	−	−	−	−	−	−
H2 (ancestral)	G/C	**T**	**G**	**A**	**C**	**G**	**C**	**G**	0.45	None	None	Low	Low	Low	−	−	−	−	−	−	−	−
H2-2	C/G	**T**	**G**	**A**	T	**G**	**C**	**G**	0.03	None	None	None	Low	None	−	−	−	−	−	−	−	−
H3	C/G	**T**	**G**	T	**C**	**G**	**C**	**G**	0.38	0.53	0.77	0.73	0.74	0.54	-0.045	0.012	2.0E-4	0.944	0.889	0.027	9.3E-6	0.685
H3-1	G	C	**G**	T	**C**	**G**	**C**	**G**	Low	0.02	None	Low	Low	0.02	−	−	−	−	−	−	−	−
H3-3	C	**T**	**G**	T	**C**	**G**	**C**	A	None	0.02	0.01	0.01	0.01	0.04	0.025	0.045	0.574	0.154	1.066	0.107	0.555	0.795
H3-4	C	**T**	**G**	T	**C**	**G**	T	**G**	None	None	0.02	0.03	0.01	None	−0.010	0.035	0.777	0.377	0.934	0.074	0.358	0.349
H4	C/G	**T**	**G**	T	T	**G**	**C**	**G**	0.07	0.01	0.03	0.03	0.05	0.07	−0.048	0.028	0.089	0.312	0.881	0.067	0.057	0.549

**Figure 2:**
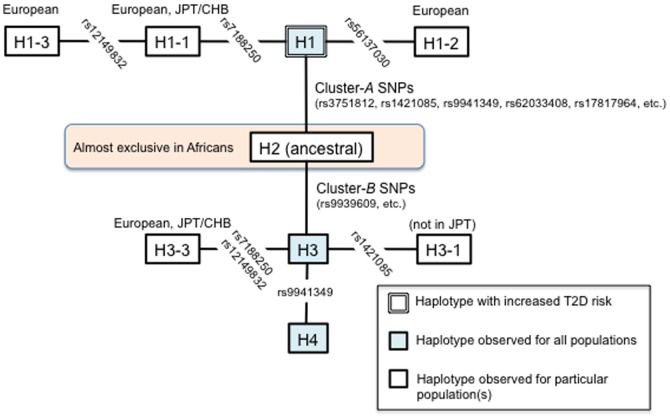
Estimated haplotype phylogeny at the *FTO* locus. Haplotypes with frequencies ≥0.05 are demonstrated in the figure, apart from H1-1, H1-2, H1-3, H3-1 and H3-3, which could be generated by recombination. Also see Table 2 and Table S5.

It is assumed that a cluster of base-substitutions (involving rs1421085, rs3751812, rs9941349, rs56137030, rs17817964, and rs12149832) initially occurred, followed by another cluster of base-substitutions (involving rs9939609) within the target interval at *FTO*. Then, presumably the underlined alleles were produced by either recombination or recurrent base-substitutions that occurred independently at each SNP (see Figure 2 and Table S5). Ancestral types of alleles are shown in bold letters.

In the estimates of haplotype frequency, they are labelled with “Low”when the frequency is <0.004 (except for H1–2 in the European-ancestry population).

There is recombination between rs7206790 and other assayed SNPs. For rs7206790, haplotypes with recombination are further split by two alleles; the major and minor alleles are shown in the left and right sides of the slash for the corresponding haplotypes.

rs3751812 was genotyped in T2D-association samples alone (but not in BMI-association samples) because rs3751812 and rs9939609 could not be differentiated due to the absence of H2 haplotype class in populations of East Asian and European ancestry. BMI/T2D association in each population was calculated by using haplotype-based linear/logistic regression with PLINK. The effect sizes of three Asian populations (Japanese, Vietnamese and Sri Lankan) were combined by a fixed-effect meta-analysis, while SriLankan data were not used for H1-3 and H3-4 because of the low frequency.

*Besides the SNPs tagging individual LD clusters that were drawn from T2D association results (see Figure 1), rs1421085 and rs56137030 were characterized in the present study, because they were reported to be promising candidates for causal variants at *FTO* in African American populations [7].

**Test of heterogeneity in effect size between the three populations meta-analyzed in the present study.

From the index SNPs, four principal haplotypes (H1–H4) could be inferred across the ethnic groups; rs7206790 was not used for the current haplotype analysis, since there was recombination between rs7206790 and the others (Table 2). H2 was an ancestral haplotype and almost exclusively detectable in populations of African ancestry. The phylogeny of haplotypes indicated that H3 and H4 were successively derived from H2, while H1 was derived from H2 independently (Figure 2). In the historical context, H1 was generated by a cluster of SNPs (including rs3751812; cluster *A*) and H3 was generated by another cluster of SNPs (including rs9939609; cluster *B*); these two haplotypes (H1 and H3) appeared to have occurred at different times. That is, from the inferred haplotypes, base-substitutions in the cluster *A* were likely to have initially occurred, followed by base-substitutions in the cluster *B* within the target interval at *FTO*. Then, a subset of H1 and H3 haplotypes (H1-1∼H1-3 and H3-1∼H3-4) and H4 were generated presumably by either recombination or recurrent base-substitutions for SNPs in the cluster *A*, which occurred independently at each SNP (Figure 2, Table 2 and Table S5). Accordingly, the phylogeny of haplotypes became appreciably complex and some of the cluster-*A* SNPs tagged >1 haplotypes at *FTO*; for example, rs9941349 tagged H4 in addition to H1.

The H1 and H3 haplotypes showed significant associations with opposite effects on the trait (i.e., BMI and diabetes-risk elevation for H1; BMI and diabetes-risk reduction for H3) in the direction consistent among the three Asian populations (Table 2). Since the two clusters of SNPs, each tagging H1 and H3, respectively, were in LD (*r^2^*>0.5) to each other in non-African populations (Figure S2) and the two haplotypes were located closely in the phylogeny (Figure 2), the negative effect of H3 was considered to reflect a “mirror image” of BMI and diabetes-risk elevating alleles for the cluster-*A* SNPs tagging H1 (Table 2 and Table S6). A replication study in Chinese confirmed significant (*p* = 2.0×10^−4^) association between H1-haplotype and BMI and also supported consistency in the direction of association between the H1-haplotype and type 2 diabetes (Table S7). Taken together, the H1 haplotype turned out to exert the most significant effect on both phenotype traits, and the SNPs tagging H1 were most correlated with the causal variant(s) at *FTO* in Asians.

### Filtering of SNPs by trans-ethnic comparison

Recently, meta-analysis involving >20,000 African American subjects was conducted [7], reporting a series of genetic variants with significant BMI association at *FTO*; the variants were found to be exactly among the cluster-*A* SNPs in the present study (Figure S4).

**Figure 3:**
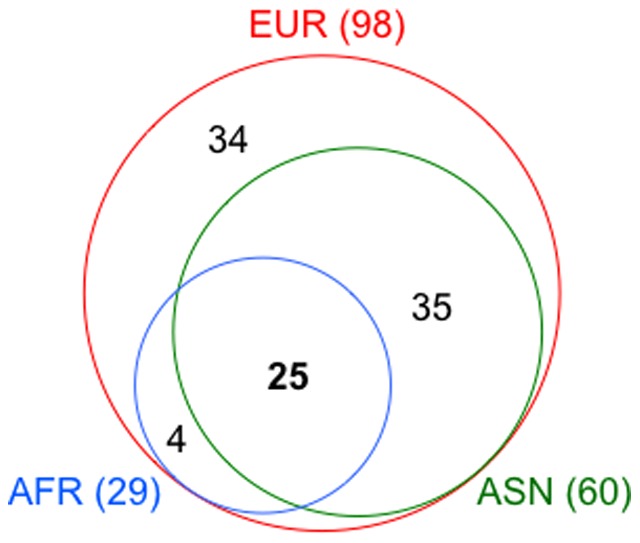
Overlap of SNPs associated with BMI at *FTO*. The Venn diagram illustrates the number of SNPs that show association with BMI in any of three ethnic groups. The total number of associated SNPs in individual ethnic groups is listed in parentheses after the ethnic group name.

As an approach for further reducing the list of potential causal variants, we utilized inter-ethnic diversity in LD patterns as well as in the SNP–trait associations at *FTO*. Among those showing significant association (LD clusters 1–7) in Europeans (Figure 1), SNPs in LD clusters 1 and 2 did not appear to show BMI association in African Americans. In addition, SNPs in LD clusters 2 and 5 were excluded from the list of potential causal variants in Asians based on the conditional and haplotype-based association analyses (Table 1, Table S3, Table S6 and Table S7). Considering these findings, we generated a list of SNPs correlated with index SNPs, which were pertinent to each of three major ethnic groups—European (EUR), Asian (ASN) and African (AFR) ancestries in the 1000 Genomes Project data, and then examined the inter-ethnic-group overlapping (Table S8). Thus, we identified a total of 25 SNPs to overlap between the ethnic groups and considered that these should contain the causal variant(s) (Figure 3). While 4 SNPs turned out to be overlapped between EUR and AFR but not with ASN (Table S8), one of them (rs9941349) was not previously reported [7] because of its failure to attain a given cut-off level, thus constituting the difference in the target SNP list between the current study and the African study as discussed below.

## Discussion

We conducted fine-scale mapping of genetic association with BMI and type 2 diabetes in the intron-1 *FTO* region and successfully narrowed the list of target variants to 25 SNPs, which are likely to contain the causal variant(s) shared across the populations. In this process, we integrated results from multiple ethnic groups by partitioning of associated variants into LD clusters and trans-ethnic filtering of them. To our knowledge, this is the first study to demonstrate that the power to resolve the causal variants from those in strong LD increases consistently when three distant populations—Europeans, Asians and Africans—are included in the follow-up study, in accordance with the previous simulations [3].

Fine mapping was recently conducted in African Americans for BMI association at the 16q12.2/*FTO* locus [7] and showed that the number of target SNPs was 28, which appeared to be much smaller than that previously indicated in Europeans [19], [20]. Although the difference in number has turned out to be relatively small (i.e., 28−25 = 3), the present study further provides two lines of evidence for *FTO* association; (1) the actual presence of shared (or universal) causal variant(s), which has been confirmed by trans-ethnic comparison of association signals at single SNP- and haplotype-levels and (2) the increased power for fine-mapping resolution by the addition of Asians to Europeans and Africans.

The basic concept underlying trans-ethnic fine mapping is thought to be that the LD between the observed (or tag) SNPs showing association and the unknown causal variant(s) is similar across different populations even if the degree of LD conservation in their vicinity could differ substantially in the patterns of SNP correlation and the lengths of haplotypes. This will enable us to identify the same causal variant(s) underpinning validated association signals across the populations. As a number of fine mapping studies on other complex disease/trait loci have indicated [1], [21], it is possible that there are >1 causal variants for a given locus and these variants are population-specific, at least, in part. Therefore, we should first verify that the same SNPs and their proxies show significant and independent associations reproducibly among the populations, which can be then used for trans-ethnic fine mapping. Recognizing that there are some limitations in using the imputed genotypes from the SNP coverage of the existing GWAS chips in terms of accuracy, we have found our approach of partitioning the associated variants into LD clusters to be useful for the subsequent conditional and haplotype analyses in the situation, where a large number of SNPs display statistical signals of similarly strong magnitude and such a highly conserved LD does not allow for the functional SNP to be conveniently distinguishable.

The stage of conditional analysis seems to be analogous to some reported methods, such as the one recently used for fine mapping of the *FGFR2* breast cancer risk locus [21]. Briefly, with a custom dense-genotyping chip, which was designed to have a high coverage of SNPs in the target region (500 kb in size), the whole study subjects (*n* = 89,050 from three ethnic groups) were initially characterized; the assayed genotype data were then used to estimate genotypes for other common variants across the region by imputation with the 1000 Genomes Project data set. Among a total of 2,729 SNPs (438 assayed and 2,291 well-imputed SNPs), 392 SNPs attained *p*<1×10^−4^ and were subjected to conditional analysis, by which three independent association signals were identified in Europeans and one of them reproduced in Asians. In the end, statistical fine mapping at *FGFR2* could narrow the number of candidate variants to 8 by excluding the SNPs that were correlated with but less significant than the strongest SNP for each of the association signals, primarily based on the European data.

The present study, on the other hand, used the genotype data assayed by GWAS chips and imputed them to the HapMap Phase 2 and 1000 Genomes Project data sets in Japanese as well as the summary data for the published European GWAS. Although dense genotyping was not performed in the Japanese GWAS subjects, LD clusters have turned out to be sufficiently definable from the tightly correlated SNPs (*r^2^*≥0.56 in East Asians; Figure S2) in the target region of *FTO*. The absence of additional LD cluster is also supported by a list of target SNPs, which were identified with a custom dense-genotyping chip in African Americans; that is, these target SNPs were all partitioned into any of LD clusters 3–6 defined in Asians and Europeans (Figure S4). In addition, of note is the fact that the list of associated SNPs is identical for European GWAS between BMI and type 2 diabetes (Figure 1), suggesting that the same causal variant(s) should underpin association signals for both phenotypes in the target region. Subsequent conditional analysis based on LD clusters has allowed to reduce the possible list of common causal variants (Table S3). Accordingly, there is some advantage to the current approach in verifying that the same SNPs and their proxies, which together form the individual LD clusters, show significant and independent associations reproducibly across the populations of European and non-European ancestry, using GWAS data, without additional dense genotyping.

Trans-ethnic comparison of association signals at a haplotype level has shown that the H1 haplotype exerts the most significant effect on BMI and type 2 diabetes (Table 2), indicating that the cluster-*A* SNPs tagging H1 are most correlated with the causal variant(s) at *FTO*. The phylogeny of haplotypes can clearly account for the repeatedly argued discrepancy of rs9939609–trait association between Africans and non-Africans [6], [7]. This is largely due to the inter-population differences in the H2-haplotype frequency; common (45%) in Africans and almost null in non-Africans (Table 2, Table S6 and Table S7). Furthermore, the haplotype analysis has revealed that some of the cluster-*A* SNPs tag >1 haplotypes at *FTO* in a population-specific manner, thereby making the phylogeny of haplotypes appreciably complex (Figure 2 and Table 2). While genetic impacts of such population-specific haplotypes (e.g., H1-2 in Europeans) on the traits remain undefined, we cannot exclude the SNPs tagging the population-specific haplotypes from the list of potential causal variants.

Besides incorporating the data on both conditional and haplotype analyses into fine mapping, the current approach has filtered SNPs through trans-ethnic comparison (Table S8). Although reasonably reduced by our fine mapping, the list of 25 SNPs is still large for follow-up functional evaluation. Recently, to make functional assignment of regulatory information onto genomic variants, the use of integrated databases, such as the RegulomeDB [22] and HaploReg [23], has become an approach of interest. According to the RegulomeDB variant classification scheme, rs17817964 has attained some evidence (Category 2b, which is the most confident among 25 SNPs) for a variant to be located in a functional region (Table S8). According to the HaploReg, on the other hand, several other SNPs, such as rs1421085, are noted for their functional location and likely functional consequences. Taken together, although the catalog of information from the integrated databases helps to further refine the SNP list and to give priority to a subset of candidate variants, functional studies should be undertaken at some stage of fine mapping.

There are some limitations in trans-ethnic fine mapping. First, the imputation of genotypes is a powerful approach but the concordance between the assayed and imputed genotypes should be carefully assessed, in particular, in the populations of African ancestry. Second, conditional analysis may not provide sufficient power to distinguish independent association signals, unless the LD between the target SNPs is relatively decayed and an appreciably large number of samples are available for the analysis in the studied population. Third, the availability of association results for African-descent populations is, although not always, the key to the success of trans-ethnic fine mapping.

While we are preparing this manuscript, a study [24] has found compelling evidence that, rather than affecting *FTO* itself, the causal variant(s) can influence expression of a distant gene, *IRX3*, which is located around 500-kb away from the first intron of *FTO*. Although the presence of long-range interactions between the two genes has been proven by a technique called chromatin conformation capture, it remains to be determined whether the genetic mechanism involves disrupted regulation of *FTO*, *IRX3* or both, by pinpointing the true functional variant (or variants) in the target *FTO* region [25].

In summary, the current trans-ethnic fine mapping of *FTO* association has successfully narrowed the list of target variants to 25 SNPs, which are the smallest in number among the studies conducted to date for *FTO*. Also, an advantage of our systematic fine-mapping approach—partitioning of associated variants into LD clusters and trans-ethnic filtering—is the applicability to the existing GWAS data in combination with direct genotyping of selected variants. Further investigation is warranted to evaluate its usefulness at other established loci, where a highly conserved LD does not allow for the functional SNP to be conveniently distinguishable in Europeans.

## Supporting Information

Figure S1 **Regional distribution of proxy SNPs for each LD cluster in 3 ethnic groups.** Proxies to each of the index SNPs (for LD clusters 2–6) are arranged according to the chromosomal position (from 53.79 to 53.85 Mb on chromosome 16; Build 37) in HapMap CEU (**a**), JPT+CHB (**b**), and YRI (**c**). In each ethnic group, SNPs derived from the HapMap Project (top, *r^2^*≥0.9) and 1000 Genomes Project (bottom, *r^2^*≥0.95) are shown separately. In case that proxies to an LD cluster cannot be differentiated from those to another LD cluster, they are merged to either of the LD clusters with different colors, which are compatible with the LD clusters in HapMap CEU.
(PDF)

Figure S2 **Comparison of LD coefficients between ethnic groups.** Among those directly assayed in the present study, HapMap SNPs are demonstrated; rs56137030, rs62033408 and rs7188250 (1000 Genomes Project) are not included in the table. LD coefficients (*r^2^* and *D'* ) between the SNPs are calculated using data for the current study sample (in the top panels) and those for HapMap sample (in the bottom panels). N.B., rs9939609 and rs3751812 are in complete LD (*r^2^* = 1.000) in HapMap JPT+CHB and CEU. rs9923544, representing LD cluster 2, is in complete LD (*r^2^* = 1.000) with rs9941349 in HapMap JPT+CHB.
(PDF)

Figure S3 **Plots of type 2 diabetes association for the 16q12.2/** ***FTO*** **region in Japanese; genotypes are imputed to the 1000 Genomes Project data set.** SNPs in the LD block are largely partitioned into two subsets using the extent of LD with any of 4 index SNPs (rs7206790, rs62033400, rs17817964 and rs12149832) each representing LD clusters 1, 3, 4 and 6, which are associated with the traits in Japanese. Blue squares, SNP with 0.8≤*r^2^*≤1.0 to the index SNPs; black squares, SNP with *r^2^*<0.8 to the index SNP in ASN (see Table S8). Also, refer to the legend for Figure 1.
(PDF)

Figure S4 **Plots of BMI association for the 16q12.2/** ***FTO*** **region in African Americans; data are drawn from the published study [7].** The associated SNPs are partitioned into clusters and colored as shown in Figure 1. rs56137030 was reported to show the strongest association signal in African American individuals and SNPs with *r^2^*>0.5 to rs56137030 are displayed in the bottom panel.
(PDF)

File S1 **File includes Tables S1–S8.** Table S1: Clinical characteristics of study participants. Table S2: SNP–type 2 diabetes association at the *FTO* locus. Table S3: Table S3A: Conditional analysis of BMI association with a pair of SNPs being simultaneously included in the regression model. Table S3B: BMI association by linear regression. Table S4: Conditional analysis of T2D association with a pair of SNPs being simultaneously included in the regression model. Table S5: *FTO* Haplotypes involving proxies (1000G phase-1 data) to index SNPs. Table S6: *FTO* Haplotypes involving index SNPs and their association with BMI and type 2 diabetes in Asian studies. Table S7: *FTO* Haplotypes involving index SNPs and their association with BMI and T2D in Chinese replication study. Table S8: A list of SNPs correlated with index SNPs for BMI/type 2 diabetes association in the *FTO* intron 1 locus.
(XLSX)

Text S1 **Supporting information on fine mapping by trans-ethnic “partition and filter” approach.**
(PDF)
