# Supplementary material for: Systematic Fine-Mapping of Association with BMI and Type 2 Diabetes at the FTO Locus by Integrating Results from Multiple Ethnic Groups
Source: PLoS One. 2014 Jun 30;9(6):e101329. doi: 10.1371/journal.pone.0101329 (PMC4076329; doi:10.1371/journal.pone.0101329)
Supplement: Figure S1 — Regional distribution of proxy SNPs for each LD cluster in 3 ethnic groups. Proxies to each of the index SNPs (for LD clusters 2–6) are arranged according to the chromosomal position (from 53.79 to 53.85 Mb on chromosome 16; Build 37) in HapMap CEU (a), JPT+CHB (b), and YRI (c). In each ethnic group, SNPs derived from the HapMap Project (top, r2≥0.9) and 1000 Genomes Project (bottom, r2≥0.95) are shown separately. In case that proxies to an LD cluster cannot be differentiated from those to another LD cluster, they are merged to either of the LD clusters with different colors, which are compatible with the LD clusters in HapMap CEU. (PDF) [file pone.0101329.s001.pdf]

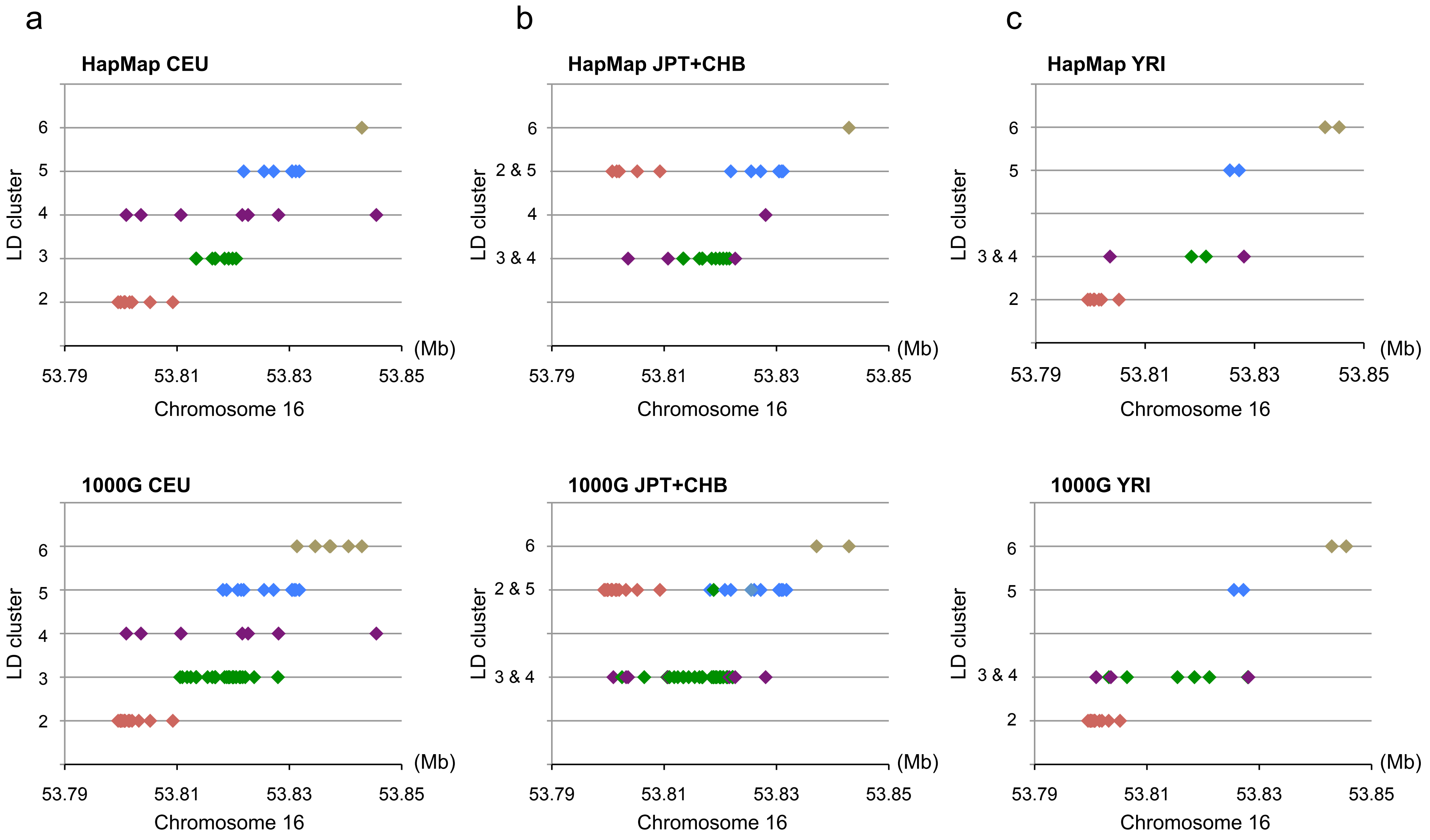

Figure S1. Regional distribution of proxy SNPs for each LD cluster in 3 ethnic groups. Proxies to each of the index SNPs (for LD clusters 2–6) are arranged according to the chromosomal position (from 53.79 to 53.85 Mb on chromosome 16; Build 37) in CEU (**a**), JPT+CHB (**b**), and YRI (**c**). In each ethnic group, SNPs derived from the HapMap Project (top, $r^2 \geq 0.9$) and 1000 Genomes Project (bottom, $r^2 \geq 0.95$) data sets are shown separately. In case that proxies to an LD cluster cannot be differentiated from those to another LD cluster, they are merged to either of the LD clusters with different colors, which are compatible with the LD clusters in CEU.

LD cluster 2: proxy to rs9923544
LD cluster 3: proxy to rs9939609 (rs3751812)
LD cluster 4: proxy to rs17817964
LD cluster 5: proxy to rs9941349
LD cluster 6: proxy to rs12149832
