# Supplementary material for: Systematic Fine-Mapping of Association with BMI and Type 2 Diabetes at the FTO Locus by Integrating Results from Multiple Ethnic Groups
Source: PLoS One. 2014 Jun 30;9(6):e101329. doi: 10.1371/journal.pone.0101329 (PMC4076329; doi:10.1371/journal.pone.0101329)
Supplement: Figure S2 — Comparison of LD coefficients between ethnic groups. Among those directly assayed in the present study, HapMap SNPs are demonstrated; rs56137030, rs62033408 and rs7188250 (1000 Genomes Project) are not included in the table. LD coefficients (r2 and D' ) between the SNPs are calculated using data for the current study sample (in the top panels) and those for HapMap sample (in the bottom panels). N.B., rs9939609 and rs3751812 are in complete LD (r2 = 1.000) in HapMap JPT+CHB and CEU. rs9923544, representing LD cluster 2, is in complete LD (r2 = 1.000) with rs9941349 in HapMap JPT+CHB. (PDF) [file pone.0101329.s002.pdf]

Vietnamese (Hanoi+Thai Binh, n=3,324)

	Effect/ other alleles	Effect allele freq.	rs7206790	rs1421085	rs9939609	rs9941349	rs17817964	rs12149832
rs7206790	G/C	0.192		0.481	0.483	0.437	0.449	0.416
rs1421085	C/T	0.188	0.225		0.991	0.994	0.995	0.952
rs9939609	A/T	0.189	0.229	0.974		0.983	0.981	0.939
rs9941349	T/C	0.237	0.146	0.737	0.725		0.906	0.929
rs17817964	T/C	0.201	0.190	0.910	0.891	0.665		0.930
rs12149832	A/G	0.192	0.173	0.887	0.869	0.657	0.813	

D′

r^2

Japanese (Amagasaki+Fukuoka, *n*=10,673)

	Effect allele freq.	rs7206790	rs1421085	rs9939609	rs9941349	rs17817964	rs12149832
rs7206790	0.212		0.841	0.824	0.681	0.665	0.762
rs1421085	0.183	0.587		0.987	0.976	0.974	0.950
rs9939609	0.184	0.570	0.964		0.979	0.974	0.952
rs9941349	0.210	0.458	0.802	0.817		0.829	0.926
rs17817964	0.216	0.432	0.770	0.780	0.664		0.925
rs12149832	0.189	0.502	0.867	0.879	0.752	0.724	

D′

r^2

Sri Lankan (Ragama+estate, n=3,029)

	Effect allele freq.	rs7206790	rs1421085	rs9939609	rs9941349	rs17817964	rs12149832
rs7206790	0.345		0.917	0.907	0.833	0.910	0.828
rs1421085	0.343	0.835		0.981	0.915	0.988	0.905
rs9939609	0.328	0.761	0.898		0.989	0.993	0.983
rs9941349	0.399	0.551	0.660	0.720		0.995	0.799
rs17817964	0.325	0.759	0.901	0.974	0.719		0.999
rs12149832	0.368	0.621	0.736	0.810	0.559	0.827	

D′

r^2

HapMap rel.27 JPT (*n*≤86)

	Effect allele freq.	rs7206790	rs1421085	rs9939609 rs3751812	rs9941349	rs17817964	rs12149832
rs7206790	0.190		0.734	0.711	0.606	0.664	0.664
rs1421085	0.188	0.539		1.000	0.906	0.912	0.914
rs9939609 rs3751812	0.186	0.462	0.915		1.000	1.000	1.000
rs9941349	0.221	0.268	0.602	0.671		0.833	0.911
rs17817964	0.182	0.441	0.765	0.842	0.560		0.917
rs12149832	0.170	0.441	0.835	0.915	0.620	0.778	

D′

r^2

HapMap rel.27 CEU (*n*≤113)

	Effect allele freq.	rs7206790	rs1421085	rs9939609 rs3751812	rs9941349	rs17817964	rs12149832
rs7206790	0.509		0.847	0.847	0.749	0.806	0.808
rs1421085	0.460	0.536		0.960	0.959	1.000	0.960
rs9939609 rs3751812	0.460	0.536	0.922		0.959	1.000	0.960
rs9941349	0.473	0.437	0.885	0.885		1.000	0.961
rs17817964	0.442	0.474	0.961	0.961	0.927		1.000
rs12149832	0.450	0.476	0.922	0.922	0.889	0.963	

D′

r^2

HapMap rel.27 YRI (*n*≤113)

	Effect allele freq.	rs7206790	rs1421085	rs3751812	rs9941349	rs17817964	rs12149832
rs7206790	0.592		1.000	1.000	0.414	1.000	1.000
rs1421085	0.066	0.038		1.000	1.000	1.000	1.000
rs3751812	0.066	0.038	1.000		1.000	1.000	1.000
rs9941349	0.173	0.044	0.339	0.339		1.000	1.000
rs17817964	0.059	0.043	1.000	1.000	0.339		1.000
rs12149832	0.050	0.036	0.825	1.000	0.280	0.849	

D′

r^2

D′ >0.95
>0.9

r^2 >0.9
>0.8

Figure S2. Comparison of LD coefficients between ethnic groups.
Among those directly assayed in the present study, HapMap SNPs are demonstrated; rs56137030, rs62033408 and rs7188250 (1000 Genomes Project) are not included in the table. LD coefficients (r^2 and *D'*) between the SNPs are calculated using data for the current study sample (in the top panels) and those for HapMap sample (in the bottom panels).
N.B., rs9939609 and rs3751812 are in complete LD (r^2 =1.000) in HapMap JPT+CHB and CEU. rs9923544, representing LD subgroup 1, is in complete LD (r^2 =1.000) with rs9941349 in HapMap JPT+CHB.
