# Supplementary figures and images for: Systematic Fine-Mapping of Association with BMI and Type 2 Diabetes at the FTO Locus by Integrating Results from Multiple Ethnic Groups
Source: PLoS One. 2014 Jun 30;9(6):e101329. doi: 10.1371/journal.pone.0101329 (PMC4076329; doi:10.1371/journal.pone.0101329)

Figure S3

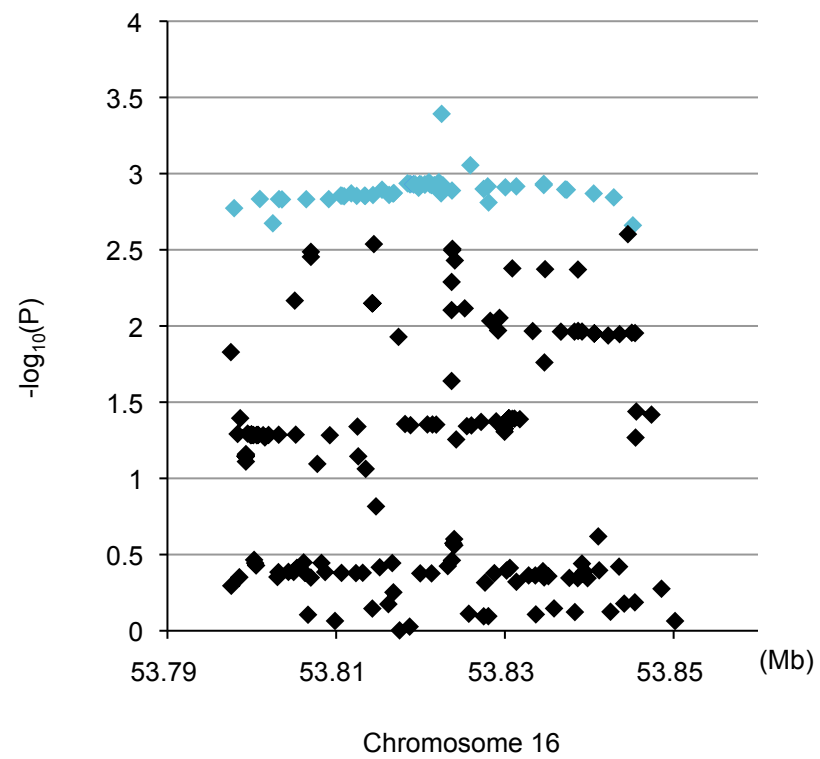

Supplement: Figure S3 — Plots of type 2 diabetes association for the 16q12.2/ FTO region in Japanese; genotypes are imputed to the 1000 Genomes Project data set. SNPs in the LD block are largely partitioned into two subsets using the extent of LD with any of 4 index SNPs (rs7206790, rs62033400, rs17817964 and rs12149832) each representing LD clusters 1, 3, 4 and 6, which are associated with the traits in Japanese. Blue squares, SNP with 0.8≤r2≤1.0 to the index SNPs; black squares, SNP with r2<0.8 to the index SNP in ASN (see Table S8). Also, refer to the legend for Figure 1. (PDF) [file pone.0101329.s003.pdf]
