# Supplementary material for: Systematic Fine-Mapping of Association with BMI and Type 2 Diabetes at the FTO Locus by Integrating Results from Multiple Ethnic Groups
Source: PLoS One. 2014 Jun 30;9(6):e101329. doi: 10.1371/journal.pone.0101329 (PMC4076329; doi:10.1371/journal.pone.0101329)
Supplement: Figure S4 — Plots of BMI association for the 16q12.2/ FTO region in African Americans; data are drawn from the published study [7]. The associated SNPs are partitioned into clusters and colored as shown in Figure 1. rs56137030 was reported to show the strongest association signal in African American individuals and SNPs with r2>0.5 to rs56137030 are displayed in the bottom panel. (PDF) [file pone.0101329.s004.pdf]

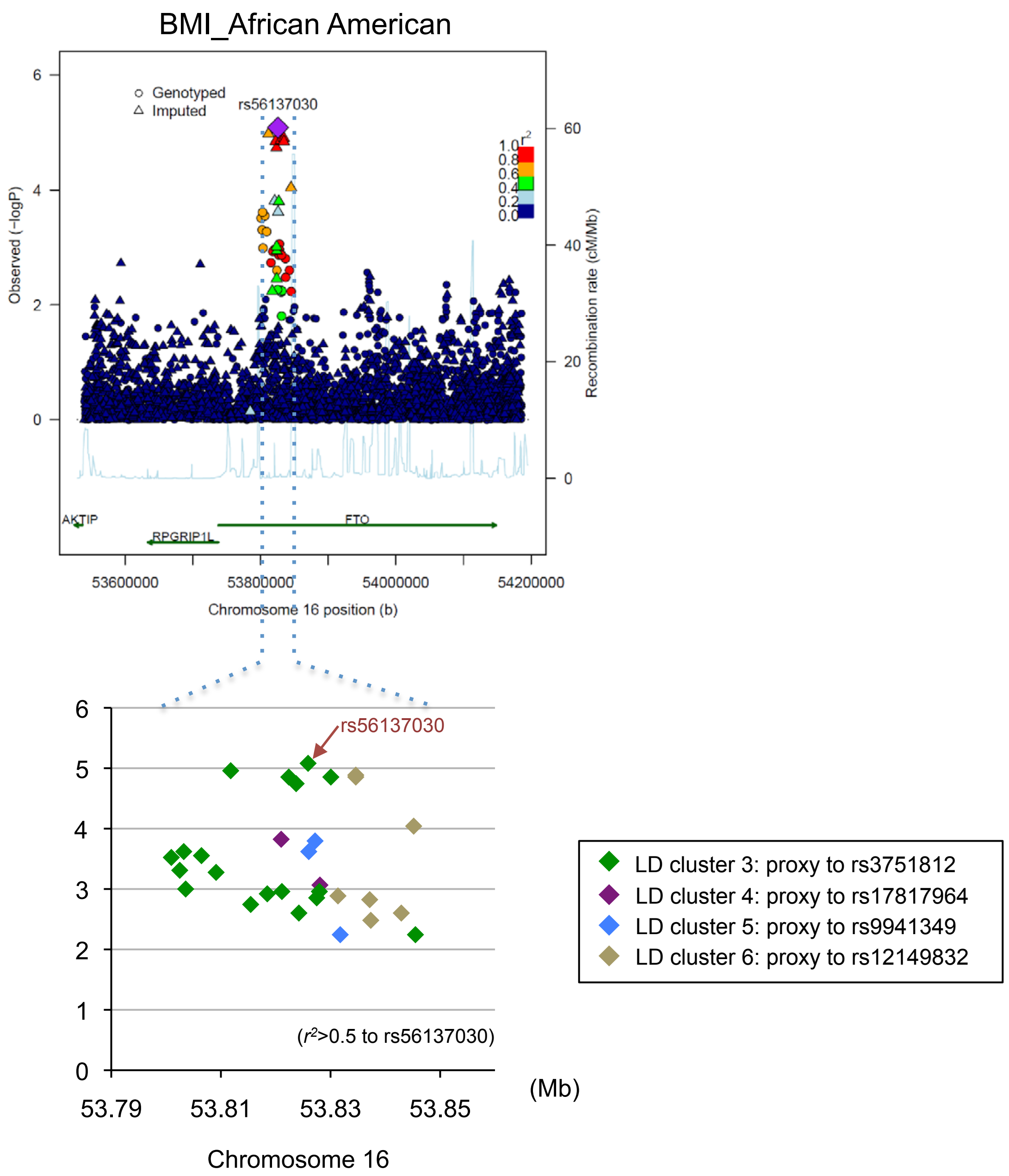

Figure S4

Plots of BMI association for the 16q22.2/*FTO* region in African Americans; data are drawn from the published study [7]. The associated SNPs are partitioned into clusters and colored as shown in Fig. 1. rs56137030 was reported to show the strongest association signal in African American individuals and SNPs with r^2>0.5 to rs56137030 are displayed in the bottom panel.
