# Supplementary material for: Systematic Fine-Mapping of Association with BMI and Type 2 Diabetes at the FTO Locus by Integrating Results from Multiple Ethnic Groups
Source: PLoS One. 2014 Jun 30;9(6):e101329. doi: 10.1371/journal.pone.0101329 (PMC4076329; doi:10.1371/journal.pone.0101329)
Supplement: Text S1 — Supporting information on fine mapping by trans-ethnic “partition and filter” approach. (PDF) [file pone.0101329.s006.pdf]

Supplementary Information

Fine mapping by trans-ethnic "partition and filter" approach

Tracing SNP markers to a causal variant via fine mapping is expected to increase the strength of association to a certain extent. However, if the GWAS have already evaluated a reasonably dense set of SNPs, the gains in statistical significance may not be striking. Under these circumstances, we consider several steps in our fine-mapping strategy as previously reported (Takeuchi et al. 2010). Briefly, the first step is to evaluate the strength of the quantitative trait (or disease) association at all assayed/imputed SNP markers characterized in the GWAS panel and to select a list of 'top hit' (or index) SNPs that satisfy a certain level of statistical significance, such that they belong to independent LD clusters with each other at a given LD coefficient level, e.g., r^2<0.9. In the case of *FTO*, this threshold (r^2<0.9) is applicable to our Japanese GWAS data set but not necessarily so to the HapMap JPT+CHB data set (Supplementary Fig. 2). There are modest discrepancies in r^2 values between the 2 data sets principally due to the differences in sample size. The second step is to partition all the assayed/imputed SNPs located in the target region so that those in stronger LD with one index SNP (e.g. SNP-A) than with another index SNP (e.g. SNP-B) are grouped into a bin of SNP-A's correlates. Here, the number of bins is equal to the number of selected index SNPs. The third step is to compare the strength of association at the individual index SNPs across different ethnic groups and to prioritise the ones that show the strongest association signal and its correlated SNPs (in its r^2 bin) for functional and biological follow-up of the corresponding association signal. Thus, to narrow target intervals for prioritisation in fine-mapping, we partition all the SNPs located in the tested region, exploring common causal variants by haplotype analysis.

Our systematic approach for integrating results from multiple ethnic groups consists of two parts—partitioning and filtering—as explained below.

I. Partitioning

In each population (or ethnic group), extract a list of SNPs that represent an independent association signal after the **partitioning** process as below.

A. Test the disease (or quantitative trait) association for assayed/imputed SNPs in the region of interest.

B. Define a *lead* SNP (SNP_{lead}) that shows the smallest *P*-value (P_{lead}). Include this SNP in the list at the beginning.

C. Select SNPs showing $P < 10 \times P_{lead}$ [*Note] and add them to the list.

D. Partition the SNPs in this list by testing the association in a regression model with adjustment for SNP_{lead} and refine the list of *index* SNPs [**Note]. Step I-D requires **genotype data**. If they are unavailable in a certain population, skip this step and proceed to step I-E.

   1. Remove an SNP from the list when it reflects a *secondary* association of SNP_{lead} via LD. Such an SNP and SNP_{lead} are considered to show P >0.05 and <0.05, respectively, when both are included simultaneously in the regression model.
   2. An SNP showing P <0.01 (after adjustment for SNP_{lead}) may represent an indication of *separate* association signal (i.e., allelic heterogeneity). Remove such an SNP from the list [***Note] and treat it separately for the investigation of another association signal.

E. Further select SNPs that have not been tested in steps I-A to I-D but need to be added to the list. Such SNPs should show a given level of LD with one *index* SNP, which was tested and validated in step I-D. Here, the LD coefficient should be $r^2 > r^2_{partition}$ [****Note].

F. Infer haplotypes from the genotypes of index SNPs across populations (e.g., HapMap and 1000 Genomes Project reference panels) to highlight shared

variants across ethnic groups and potential secondary signals of association.

*Note: Constraint is a practical *filter* to limit the SNPs tested in step I-D. Roughly, $qchisq(P) > r^2_{\text{partition}} \bullet qchisq(P_{\text{lead}})$. Here, *qchisq* is the quantile function of the chi-squared distribution with one degree of freedom. For the calculation of $r^2_{\text{partition}}$ see below in ****Note.

**Note: The list can be refined adaptively by increasing the sample size tested in step I-D. With the larger sample size, we can remove more SNPs from the list via partitioning in step I-D-1 and consequently we can reduce the number of *index* SNPs. Here, an *index* SNP is the one that attains the lowest *P*-value within an LD cluster (or a bin of correlates), such that *index* SNPs belong to independent LD clusters with each other at a given LD coefficient level, e.g., $r^2 < 0.9$. Also, the number of SNPs that will be added to the list in step I-E becomes smaller as $r^2_{\text{partition}}$ increases.

***Note: To perform step I for another (or second) association signal, which is identified in step I-D-2, use the association statistics after adjustment for SNP_{lead} reflecting the primary association signal.

****Note: Practically, $r^2_{\text{partition}}$ can be set at 0.9. However, the threshold can be more precisely defined according to both the sample size (of the population) available in step I-D and the strength of association at SNP_{lead}. Suppose that N_{lead} samples are necessary to detect significant association at SNP_{lead} (e.g., under the significance level of 0.05 and 80% power) while we actually have *N* samples in hand. Then, $r^2_{\text{partition}}$ is calculated to be $[1 - N_{\text{lead}}/N]$; where $r^2_{\text{partition}}$ approaches to 1 as *N* increases.

II. Filtering

Filter SNPs that have survived step I, using trans-ethnic diversity (or similarity) in

association signals among multiple populations (e.g., Japanese, Sri Lankans, and Europeans).

A. Combine the lists of SNPs (produced in step I for each population) across the populations.

B. If there is no overlap between populations, the lists are likely to represent different association signals. The non-overlapping SNPs may be specific to the individual populations. Alternatively, in case that there exists allelic heterogeneity, SNPs representing separate association signals may not overlap with each other.

C. If there is some overlap between populations, the overlapping SNPs are considered to constitute a *filtered* list. Here, SNPs included in such a list must be in strong LD to each other [due to the criteria set for LD (****Note in step I-E)] within each population, also consistent across the populations, where the relevant association has been concordantly detected.

Required data

- Steps I-A to I-C require summary association results for assayed/imputed SNPs in the regions of interest.
- Step I-D requires **genotype data**. If unavailable, this step can be skipped (in some populations).

It has to be noted that at present we focus on common (causal) variants, which are assumed to be shared across the populations; that we have not looked into the possibility of rare causal variants or common causal variants unique to one ethnic group, or the possibility that causality itself is not shared between the tested populations despite similarly positive association at the relevant locus.
